# Risk factors for adverse drug reactions associated with clopidogrel therapy

**DOI:** 10.1515/med-2021-0371

**Published:** 2022-04-07

**Authors:** Snezana Mugosa, Ivan Radosavljevic, Majda Sahman, Natasa Djordjevic, Zoran Todorovic

**Affiliations:** Department of Pharmacology, Faculty of Medicine, University of Montenegro, 81000 Podgorica, Montenegro; Clinical Trials Department, Institute for Medicines and Medical Devices of Montenegro, 81000 Podgorica, Montenegro; Department of Surgery, Faculty of Medical Sciences, University of Kragujevac, 34000 Kragujevac, Serbia; Department of Pharmacology and Toxicology, Faculty of Medical Sciences, University of Kragujevac, 34000 Kragujevac, Serbia; Department of Pharmacology, Clinical Pharmacology and Toxicology, Faculty of Medicine, University of Belgrade, Belgrade, Serbia; University Medical Center “Bežanijska kosa”, Belgrade, Serbia

**Keywords:** clopidogrel, adverse drug reactions, pharmacogenetics

## Abstract

This study aimed to investigate the possible influence of genetic and non-genetic factors on the incidence of clopidogrel adverse drug reactions (ADRs) in cardiology patients, including the most important *CYP2C19* alleles, namely *2 and *17, as well as compliance, dose, drug interactions, and clinical factors. A total of 102 clopidogrel-treated adult Caucasian patients hospitalized at the Cardiology Department of the Clinical Center of Montenegro were enrolled in the study. Data on clinical outcomes of interest were obtained by intensive monitoring ADRs during hospitalization and one year after hospital discharge. Genotyping for CYP2C19*2 and *17 was conducted using the real-time polymerase chain reaction method. ADRs were characterized using the Rawlins and Thompson classification and the World Health Organization criteria. Causality was assessed using the Naranjo probability scale. ADRs to clopidogrel were observed in 9 of 102 patients (8.8%). The observed frequencies of CYP2C19*2 and *17 were 13.2 and 25.5%, respectively. Our study, which is the first to report the frequency of *CYP2C19* polymorphism in the Montenegrin population, as well as to link the pharmacovigilance of clopidogrel with *CYP2C19* gene variability, shows that the incidence of ADRs of clopidogrel in cardiac patients is high and depends on *CYP2C19* polymorphisms, comedication/drug interactions, and gastrointestinal comorbidity.

## 1 Introduction

Clopidogrel is still used as an antiplatelet drug in a large number of cardiac patients. The safety of antiplatelet drugs should be reevaluated since it might have a significant impact on clinical practice. Data on antiplatelet agents’ relative safety are very complex to interpret and vary depending on several factors.

A study by the Italian Pharmacovigilance Network in 2017 revealed that clopidogrel seemed to be the most dangerous antiplatelet drug in Italian patients between 2009 and 2016; the total number of suspected adverse drug reactions (ADRs) reported was the following: clopidogrel (3,298) > ticlopidine (1,169) > ticagrelor (471) > prasugrel (126). However, if the rate of ADRs per the number of patients treated was compared, the rank of safety risk was different: ticagrelor > prasugrel = clopidogrel > ticlopidine [1]. Common ADRs of clopidogrel in patients include bleeding at the puncture site, hematoma, epistaxis, bruising, and gastrointestinal disorders such as gastrointestinal hemorrhage, diarrhea, abdominal pain, and dyspepsia [2]. Bleeding is the most commonly reported ADR in clinical studies and during post-marketing surveillance. In the CAPRIE study, the total incidence of any bleeding was 9.3% [3]. A recently published meta-analysis by Italian authors showed that the benefit/risk ratio for clopidogrel is more favorable than for the significantly more expensive drugs such as prasugrel and ticagrelor in patients on oral anticoagulant therapy for atrial fibrillation after a recent percutaneous coronary intervention [4]. In this cohort, the risk of clinically significant bleeding was higher if they had received ticagrelor or prasugrel rather than clopidogrel, without any additional benefit in reducing the frequency of major adverse cardiovascular events (MACE).

The interindividual variability in response to clopidogrel could be due to genetic factors (single nucleotide polymorphism of genes encoding for CYPs and P-glycoproteins) and nongenetic issues, including compliance, dosing regimen, drug interactions, and comorbidities [5]. In various studies, which included healthy volunteers of Caucasian and Asian ethnicities, a significant relationship among *CYP2C19* genotype, differences in the pharmacokinetics of clopidogrel and its active metabolite, and antiplatelet response to clopidogrel has been established [6–8]. *CYP2C19* is the most important enzyme in the metabolism of clopidogrel, which is a pro-drug because it participates in both stages of drug biotransformation to the active metabolite [6]. The gene that encodes has more than 14 allelic forms [6]. The enzyme encoded by *1 isoform normally metabolizes the drug, while the enzyme encoded by *2 isoform slowly metabolizes the drug, thus there is no expected therapeutic effect of the drug in patients with such isoforms after taking clopidogrel in usual doses (since the conversion of clopidogrel to the active metabolite by metabolism via cytochrome P450 is required for the achievement of the therapeutic effect of the drug). The CYP2C19*2/*2 allelic variant showed a strong correlation with a weaker response to clopidogrel, especially in patients younger than 45 years of age, which is considered to be the main cause of poor response to the therapy [9]. Patients with the CYP2C19 *17 isoform metabolize the drug rapidly and therefore have an increased incidence of clopidogrel ADRs [10]. Individualized clopidogrel treatment could optimize its benefit–risk ratio by reducing the risk of MACE and minimizing ADRs, such as bleeding.

This study aimed to investigate the possible influence of genetic (*CYP2C19* allelic variants *2 and *17) and nongenetic factors (noncompliance, dose, drug interactions, clinical factors) on the incidence of clopidogrel ADRs in cardiology patients.

## 2 Methods

### 2.1 Study population

A prospective study involved 102 clopidogrel-treated adult Caucasian patients hospitalized at the Cardiology Department of the Clinical Center of Montenegro, Podgorica, Montenegro. Around half of them (54 of 102) were on clopidogrel treatment before admission to the hospital, while others were prescribed clopidogrel at the time of discharge from the hospital. Patients younger than 20 years or older than 80 years, those who were on anticoagulant therapy, had hematological disorders (hemoglobin < 100 g/L, platelet count < 100 × 10^9^ cells/L or > 600 × 10^9^ cells/L), malignant or chronic inflammatory diseases, pregnant women, nursing mothers, people with dementia, and patients who were not willing to sign the informed consent form, were not included in the study.

### 2.2 Definition and classification of ADRs

Data on clinical outcomes of interest were obtained by *intensive monitoring* of ADRs: (a) inperson, during hospitalization, and (b) by telephone survey during one year after hospital discharge.

ADRs were defined and characterized according to the World Health Organization (WHO) and the Rawlins and Thompson classification. The causality relationship between the drug and the effect was established by using Naranjo’s ADR probability scale. ADRs were classified by Meyboom et al. as type A (“drug actions”), type B (“patients reactions”), and type C (“statistical”), while their severity was assessed as per the WHO criteria [11–15].

The level of intervention was attributed using a four four-level scale (Level 1 – no change in the treatment; Level 2 – dose adjustment or drug stop, no additional treatment required; Level 3 – dose adjustment or drug stop, additional treatment required; Level 4 – transfer to intensive care unit) [16]. Each ADR was also classified according to the system organ class, (according to MedDRa classification of ADRs), as recommended by the WHO [17].

### 2.3 Polymerase chain reaction (PCR) measurements

For *CYP2C19* genotyping, DNA was extracted from whole blood samples using PureLink® genomic DNA kit (Invitrogen, Carlsbad, CA). The DNA concentration was measured by the fluorometer Qubit® (Invitrogen, Carlsbad, CA), using the Qubit™ dsDNA HS Assay Kit (Invitrogen, Carlsbad, CA), and ranged from 2.4 to 24 μg/mL. Isolation of DNA was performed at the Department of Pharmacology and Toxicology, Faculty of Medical Sciences, University of Kragujevac, Serbia. Based on available literature and *CYP2C19* allele nomenclature, for the study, two variants of *CYP2C19,* namely rs4244285 (19 154G > A, CYP2C19*2) and rs12248560 (−806C > T, CYP2C19*17), were chosen to be included in the analysis as the most common genetic determinants of slow and rapid metabolizers, respectively [18,19]. Genotyping was conducted at the Department of Immunology, Public Health Institute, Kragujevac, using real-time PCR method and predesigned TaqMan^®^ Genotyping Assays C__25986767_70 and C____469857_10 (Applied Biosystems, Foster City, CA) for CYP2C19*2 and *17, respectively. The PCRs were performed in 20 ul reaction volume on SaCycler-96 RUO (SACACE Biotechnologies, Como, Italy), and the conditions constituted of initial denaturation at 95°C for 10 min, followed by 40 cycles of denaturation at 95°C for 15 sec and annealing at 60°C for 1 min.

### 2.4 Statistics

Statistical data analysis was performed using IBM SPSS Statistics 22 (SPSS Inc., Chicago, IL, USA). Descriptive statistical methods (arithmetic mean with standard deviation, and median with (interquartile) range) and methods for testing statistical hypotheses (*t*-test, Mann–Whitney test, chi-square test, and Fisher’s test of exact probability) were used for the analysis of primary data. Statistical hypotheses were tested at the level of statistical significance (alpha level) of 0.05.

The study was conducted in accordance with the Declaration of Helsinki [20], with the approval of the Ethics Committee of the Clinical Center of Montenegro and the written consent of each of the participants. Also, the study was conducted in accordance with the International Conference on Harmonisation Guideline for Good Clinical Practice [21].

## 3 Results

Baseline clinical characteristics and demographics of the study population are presented in Table 1.

**Table 1:** Demographic and clinical characteristics of the cardiac disease patients involved in the study

	Total (*N* = 102)	Patients with ADRs (*N* = 9)	Patients without ADRs (*N* = 93)	*p*-value
**Patients characteristics**				
Age, mean ± sd	59.3 ± 9.1	56.7 ± 7.1	59.6 ± 9.2	0.363
Sex, *N* (%)				
Males	70 (68.6%)	5 (55.6%)	65 (69.9%)	0.456
Females	32 (31.4%)	4 (44.4%)	28 (30.1%)	
BMI, mean ± sd	28.0 ± 3.6	26.4 ± 3.6	28.1 ± 3.6	0.161
Smoking, *n* (%)	45 (44.1%)	4 (44.4%)	41 (44.1%)	1.000
Co-morbidity type, *N* (%)	34 (33.3%)	5 (55.6%)	29 (31.2%)	0.156
– Endocrine system	8 (7.8%)	0 (0.0%)	8 (8.6%)	1.000
– Respiratory system	3 (2.9%)	0 (0.0%)	3 (3.2%)	1.000
– **Gastrointestinal system**	19 (18.6%)	5 (55.6%)	14 (15.1%)	**0.010**
– Central nervous system	5 (4.9%)	0 (0.0%)	5 (5.4%)	1.000
– Urinary system	3 (2.9%)	0 (0.0%)	3 (3.2%)	1.000
**Number of co-morbidities**				0.188
0, *N* (%)	68 (66.7%)	4 (44.4%)	64 (68.8%)	
1, *N* (%)	30 (29.4%)	5 (55.6%)	25 (26.9%)	
2, *N* (%)	4 (3.9%)	0 (0.0%)	4 (4.3%)	
**Risk factors for coronary artery disease**				
Arterial hypertension, *N* (%)	75 (73.5%)	7 (77.8%)	68 (73.1%)	1.000
Diabetes mellitus, *N* (%)	31 (30.4%)	1 (11.1%)	30 (32.3%)	0.270
Hypercholesterolemia, *N* (%)	47 (46.1%)	5 (55.6%)	42 (45.2%)	0.729
Heredity, *N* (%)	38 (37.3%)	3 (33.3%)	35 (37.6%)	1.000
Obesity, *N* (%)	21 (20.6%)	2 (22.2%)	19 (20.4%)	1.000
Number of risk factors for coronary artery disease, median (range)	3 (0–6)	3 (1–4)	3 (0–6)	0.942

Bold values indicate statistically significant value.

During the hospitalization period and 12 months after discharge from the hospital, ADRs to clopidogrel were observed in 9 of 102 patients (8.8%).

The median number of drugs used by patients with and without ADRs was 8 (range: 4–16) and 6 (range: 4–10), respectively (*p* = 0.027). Concomitant use of other drugs that are also metabolized through CYP2C19 was a significant risk factor for developing ADRs to clopidogrel (*p* = 0.030). These drugs include, for example, pantoprazole (28.4%), diazepam (13.7%), warfarin (1.9%), omeprazole (1.9%), and gliclazide (1.9%). The most commonly used pharmacotherapeutic groups of drugs in patients with comedication were antiplatelet drugs (18.7%), ACE inhibitors (11.9%), beta-blockers (9.5%), diuretics (9.3%), and statins (9.2%). The drugs that cause ADRs in patients most often belong to the following pharmacotherapeutic groups: beta-blockers (20.0%), ACE inhibitors (12.0%), organic nitrates (12.0%), anticoagulants (12.0%), and antiplatelet drugs (8.0%). The incidence of ADRs did not differ between males and females; patients with and without comorbidities, and those with and without risk factors for coronary artery disease had ADR. However, a higher percentage of patients with ADRs had gastrointestinal comorbidities than patients without ADRs (Table 1).

The distribution of *CYP2C19* nucleotide changes, haplotypes, and genotypes is shown in Table 2.

**Table 2:** Frequency of *CYP2C19* nucleotide changes, haplotypes, and genotypes

	Frequency	95% confidence interval
Nucleotide changes		
19 154G > A	0.129 (27/204)	0.092, 0.186
−806C > T	0.248 (52/204)	0.200, 0.319
Haplotype		
CYP2C19*1	0.613 (125/204)	0.544, 0.677
CYP2C19*2	0.132 (27/204)	0.092, 0.186
CYP2C19*17	0.255 (52/204)	0.200, 0.319
Genotype		
CYP2C19*1/*1	0.324 (33/102)	0.241, 0.407
CYP2C19*1/*2	0.167 (17/102)	0.106, 0.242
CYP2C19*1/*17	0.412 (42/102)	0.321, 0.496
CYP2C19*2/*17	0.098 (10/102)	0.053, 0.165

**Figure 1:**
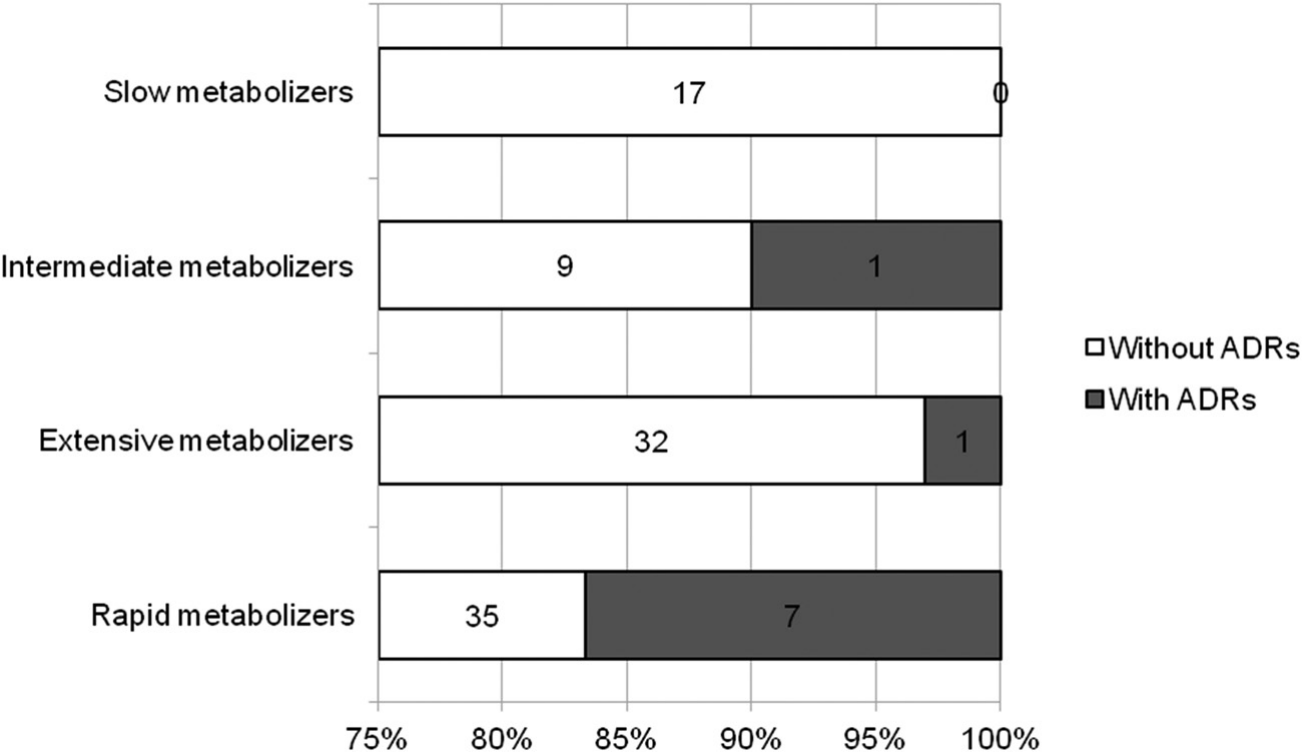
Distribution of ADRs to clopidogrel according to the CYP2C19 enzyme activity. Numbers inside the bars indicate participants without and with ADRs to clopidogrel.

Rapid metabolizers (carriers of CYP2C19*1/*17 genotype) had significantly more adverse clinical outcomes compared to slow (CYP2C19*1/*2), intermediate (CYP2C19*2/*17), or extensive (CYP2C19*1/*1) CYP2C19 metabolizers (*p* = 0.03; Figure 1).

Table 3 shows the characteristics of clopidogrel ADRs.

**Table 3:** Characteristics of detected ADRs associated with clopidogrel therapy

ADRs characteristics	*n* (%)
Type	
A	9 (100%)
B	0 (0%)
C	0 (0%)
Causality*	
Certain	1 (11.1%)
Probable	5 (55.5%)
Possible	3 (33.4%)
Level of intervention	
Level 1 (no change in dose)	7 (77.8%)
Level 2 (dose changed or the drug stopped)	1 (11.1%)
Level 3 (drug stopped + additional therapy)	1 (11.1)
Level 4 (transfer to intensive care unit)	0 (0.10%)
Severity	
Serious ADR	2 (22.2%)
Non serious ADR	7 (77.8%)
Outcome	
Death	0 (0%)
Recovery with consequences	0 (0%)
Recovery without consequences	9 (100%)

*Causality was assessed using the Naranjo probability scale (WHO criteria).

*Certain ADRs were general disorders and administration site conditions (bleeding at puncture site), probable ADRs were hematological toxicities (hematuria, thrombocytopenia), and possible ADRs were respiratory, thoracic, and mediastinal disorders (epistaxis) and vascular disorders (hematoma).

All ADRs were expected, that is, described in the Summary of product characteristics for clopidogrel.

## 4 Discussion

The most important findings of our study show that the risk of clopidogrel ADRs is higher in rapid metabolizers, patients with gastrointestinal comorbidities, and those using comedications that are metabolized via CYP2C19. In addition, this is the first study reporting the distribution of the most important *CYP2C19* polymorphisms, namely *2 and *17, in Montenegrin population.

The distribution of *1/*2 genotypes in our study fits published data for the Caucasians [22]. In contrast, we found more *1/*17 phenotype carriers than expected, but it could explain a slightly higher incidence of clopidogrel ADRs in our study than in the literature [23]. In the former Yugoslavian countries, excluding newly acquired data for Montenegro, CYP2C19 *2 and *17 allele frequencies range between 11 and 15%, and between 18 and 24%, respectively [24]. These values vary considerably across Europe, with no specific gradient relative to geographic region, as observed for *CYP2D6* alleles. However, CYP2C19*2 alleles are somewhat more common in Northern and Western Europe and *17 alleles in Central Europe [24].

In addition to the high prevalence of *17 allele carriers, the relatively high incidence of clopidogrel ADRs in our study could also be explained by differences in methodology, definitions, and classifications of ADRs, algorithms for assessing the cause-and-effect relationship between drug and ADR, etc. [25]. We have included “possible” ADRs in the total frequency of ADRs, unlike, for example, Lazarou, who listed only “certain” and “probable” ADRs, so we possibly included some false-positive results [26].

Our sample mainly consists of elderly patients with different comorbidities. Age- and comorbidity-related changes in drug pharmacokinetics make patients more susceptible to ADRs, particularly to antithrombotic agents [27]. In general, age was identified as a major predictor of preventable harm from medicines [28,29]. However, in hepatogastroenterological patients, the overall risk of ADRs was not increased with advanced age, except for patients with biliary tract diseases. Therefore, more frequent clopidogrel ADRs in our patients with gastrointestinal comorbidities could be related to their age, but further analysis is needed.

The elderly also use more medications, which is also a significant risk factor for ADRs. In a study of 9,000 Italian patients, mostly over the age of 60 years, Carbonin et al. [30] showed that the incidence of ADRs increased from 1.2% in patients receiving only one drug to 10% in those receiving nine drugs, and to about 50% in patients receiving more than ten drugs. Grymonpre et al. [31] examined the prevalence of ADRs in Canadian patients over the age of 50 years and showed that the incidence of ADRs increased from 5% in patients receiving two drugs to 20% and more in patients receiving five or more drugs. Some studies have shown that patients who receive 16 different medications during hospitalization have a 40% probability of experiencing some ADRs [32]. This could probably explain our finding of the significant difference in the incidence of clopidogrel ADRs in patients with comedications. In particular, the interaction between clopidogrel and other drugs that are substrates for CYP2C19 may be an important risk predictor for ADRs, but it should be emphasized that not all such interactions are clinically important (e.g., clopidogrel + omeprazole vs clopidogrel + diazepam).

Numerous studies have shown that the female sex is a risk factor for ADRs, although there is no reliable explanation for this in the literature. Some authors believe that lower body weight and surface area and degree of glomerular filtration, as well as higher fat content, are the reason for the higher frequency of ADRs in the female population. However, sex-related differences in the safety profile of glycoprotein IIb/IIIa inhibitors could not be found in clinical trials, which is in agreement with our study [33].

As regarding the causality, we observed the highest prevalence of probable and possible ADRs, which does not differ significantly from the data obtained in similar studies, where more than 50% of reported ADRs were classified as “possible” and less than 10% as “certain” [16,34,35]. In contrast, Classen [36] describes 62% of ADRs as “certain” ADRs and 0.7% as “probable.” We found that the incidence of serious adverse reactions was 22.2% of the total number of reported ADRs. In a study conducted by the French Center for Pharmacovigilance, this percentage was 33%, and in a study by Somers et al. it reached 38% [37,38]. In some studies, this percentage is significantly lower, which is probably attributable to differences in methodology and the population covered by the study (e.g. 30).

In our study, ADRs were 100% of type A. Given the mechanism of occurrence of these types of ADRs, the prevalence we obtained was expected, although type B reactions may reach up to one-third of detected ADR [31]. Adverse reactions were most often manifested as general disorders and administration site conditions, hematological toxicities, respiratory, thoracic, and mediastinal disorders, and vascular disorders, which fits into the safety profile of clopidogrel [2].

The distribution of patients over the levels of intervention in our study is in agreement with published data. In a study conducted by Kaur et al., in 16.3% of cases, it was not necessary to change the dose of the drug; in 12% of cases, the dose was changed, while in 75.9%, additional treatment was required [39].

Many studies have shown that the percentage of preventable ADRs is high and ranges over 50% [39,40]. This can be achieved by careful assessment of the drug’s pharmacology, its safety profile, potential to interact with other drugs, pharmacological anamnesis, and its rational prescribing tailored to the individual patient’s needs [29]. Our study confirms the need for an individual approach in the use of clopidogrel, especially in those at higher risk of ADRs [41]. Last but not least, cost–utility analysis supports genotype-guided dual antiplatelet therapy with ASA and clopidogrel [42].

Finally, the main limitation of our research is the small sample size. Additional analysis on a larger sample is necessary to resolve some issues related to the influence of comorbidities and pharmacogenetics on the safety profile of clopidogrel (e.g., P-glycoprotein-coding gene polymorphism).

## 5 Conclusion

Our study showed that the incidence of ADRs of clopidogrel in cardiac patients is high, and depends on *CYP2C19* gene polymorphisms, comedication/drug interactions, and gastrointestinal comorbidity. It is necessary to conduct continuous education of cardiologists in the safe use of drugs to improve their awareness of the ADRs of antiplatelet therapy and the related risk factors.

Our study, conducted according to the internationally accepted methodology, is the first to link the pharmacovigilance of clopidogrel and the pharmacogenetics of gene polymorphisms for *CYP2C19* in the Montenegrin population. Further investigation in a large cohort is needed to clarify the still unresolved issues regarding the individualization of therapy and the safety profile of clopidogrel and other antiplatelet agents.
